# An S-Infused/S, F-Codoped PVDF-Derived Carbon as a High-Performance Anode for Sodium-Ion Batteries

**DOI:** 10.3390/ma18174018

**Published:** 2025-08-27

**Authors:** Jianjiao Wang, Qian Zhang, Pengyu Han, Jiakun Luo, Kui-Qing Peng

**Affiliations:** Key Laboratory of Multiscale Spin Physics, Ministry of Education, Beijing Key Laboratory of Energy Conversion and Storage Materials, School of Physics and Astronomy, Beijing Normal University, Beijing 100875, China

**Keywords:** S, F-codoped carbon, anode, sodium-ion battery, PVDF-derived carbon

## Abstract

Heteroatom doping is an effective strategy for improving the sodium storage performance of hard carbon. However, the use of sulfur and fluorine codoped carbon materials as anodes for sodium-ion batteries has not been reported. Here, an S-infused/S, F-codoped PVDF-derived carbon SFC5 was prepared by one-step carbonization of PVDF and synchronously used as an anode for a sodium-ion battery. The prepared SFC5 containing 10.11 at% S and 9.54 at% F is a short-range ordered amorphous carbon with a microporous structure. Owing to the structural advantages of S, F codoping, and the high specific capacity of S, SFC5 exhibited an outstanding sodium storage performance of 365 mAh g^−1^ after 200 cycles at 50 mA g^−1^ and 212 mAh g^−1^ after 500 cycles at 400 mA g^−1^. Moreover, theoretical calculations based on density functional theory (DFT) verify that S and F codoping can considerably reduce the Na^+^ adsorption energy and increase the electronic conductivity of SFC5. The current study presents a viable and facile approach to prepare high-performance, low-cost anode materials for SIBs, supported by empirical evidence and theoretical computations.

## 1. Introduction

The demand for secondary energy storage technology is increasing with the popularization of renewable energy and high-power energy-consuming equipment, electric vehicles, etc. [1]. Electrochemical energy storage technology has attracted extensive attention because of its high energy density and low cost [2]. Lithium-ion batteries (LIBs), some of the most mature electrochemical energy storage technologies so far, have dominated the recent battery market. However, the rapid consumption of lithium resources has recently led to an increase in the cost of LIBs, resulting in their use mainly in fields requiring high-energy and high-power density batteries, such as consumer electronics and electric vehicles. Therefore, low-cost, high-performance batteries must be developed to compensate for the absence of LIBs in areas requiring low-cost and long-term cycling, such as large-scale energy storage for grids [3]. The elements sodium and lithium, which are in the same group, exhibit similar chemical properties, but sodium is more abundant and more evenly distributed in the Earth’s crust (lithium 0.006 wt% versus sodium 2.83 wt%) than lithium. Thus, sodium-ion batteries (SIBs) are ideal replacements for LIBs in the field of large-scale energy storage [4,5].

Low-cost carbon materials with diverse sources, high conductivity, and environmental friendliness are ideal candidates for use as SIB anodes [6,7]. Hard carbon (HC) materials used as anodes for SIBs exhibit a low reversible capacity because of their small interlayer spacing and finite defects. Studies have shown that appropriate heteroatom doping can dramatically enhance the sodium storage performance of HCs by expanding the interlayer spacing and introducing abundant defects and heteroatom active sites [8,9,10,11,12,13]. Ideal heteroatoms possess the following physical and chemical properties. First, the doped elements should have greater electronegativity to easily combine with carbon by covalent bonds, which can produce abundant edge defects and improve the electron conductivity of HCs. In addition, a larger atomic radius is critical to expanding the interlayer spacing of HCs, which helps improve the diffusion kinetics of ions in the electrodes [4].

Fluorine, which is the most electronegative element, is a perfect doping element in several studies; for example, CF_2_-doped reduced graphene oxide (F-rGO), fluorinated hard carbon, and fluorine-doped microcrystalline graphite, among others, exhibit enhanced sodium storage performance [14,15,16]. In addition, the strategy of enhancing the sodium storage performance by doping with the element sulfur, which has a large atomic radius, has been reported [17,18,19]. More importantly, sulfur is also a sodium storage material with a high theoretical specific capacity, whereas the use of pure sulfur as an anode for SIBs results in unsatisfactory sodium storage performance because of its lower intrinsic electron conductivity. A typical strategy to improve the sodium storage capacity of sulfur is to infuse it into a host material with high electron conductivity, such as carbon. Furthermore, on the basis of single-element doping, double-element or multi-element doping can considerably improve the electrochemical performance of secondary alkali-ion batteries by exerting synergistic effects on various doping elements. For example, the S–N codoped hollow carbon nanospheres (SN–HCSs) synthesized by Dong Quanfeng’s group, the nitrogen–fluorine codoped graphene (NFG) composite material prepared by Feng Wei’s group, and the sulfur–phosphorus codoped carbon (PSC) material synthesized by Jiang Kai et al. have shown that appropriate double-element doping can effectively improve the electrochemical performance of the corresponding secondary alkali ion batteries [20,21,22].

However, there is a dearth of research on sulfur and fluorine-codoped carbon-based materials applied to SIBs. PVDF, a fluorine-containing polymer, is an ideal precursor for preparing fluorine-doped carbon with a micropore structure [23,24], and its micropore framework is helpful to the infusion of doping elements into carbon hosts [25]. In addition, PVDF-derived carbon was widely employed as an energy storage and adsorbing material [23,25,26,27]. Therefore, in this study, an S-infused/S- and F-codoped carbon, SFC5, was synthesized as an anode for SIB by a facile one-step pyrolysis method using PVDF as both a carbon and fluorine source, and sublimed sulfur as a sulfur source, allowing the achievement of two desirable outcomes, S and F doped into carbon and S infused into the carbon host. X-ray photoelectron spectroscopy (XPS), energy dispersive X-ray spectroscopy (EDS), and thermogravimetric analysis (TGA) confirmed that S and F were successfully doped into the pyrolysis carbon of PVDF and that some of the S was present in the pores of the carbon matrix. Compared with single F-doped FC5 obtained by direct pyrolysis of PVDF and S, F-codoped FC5-S5 synthesized by a two-step method, SFC5 has an outstanding sodium storage performance of 365 mAh g^−1^ after 200 cycles at 0.05 A g^−1^ and a long cycling stability of 212 mAh g^−1^ after 500 cycles at 0.4 A g^−1^. Theoretical calculations based on density functional theory (DFT) further verified that S and F codoping can considerably improve the adsorption capacity for Na^+^ and the electron conductivity of SFC5. The method developed for the preparation of S-infused/S, F-codoped carbon materials in the current study provides a reference for the subsequent development of double- and multi-element-doped carbon materials, and the S-infused/S, F-codoped carbon materials here inspire the subsequent development of low-cost anode materials for SIBs.

## 2. Materials and Methods

### 2.1. Materials Preparation

#### 2.1.1. Preparation of the S-Infused/S, F-Codoped Carbon Material (SFC5)

PVDF and sublimed sulfur (1:2 mass ratio) were mixed homogeneously by ball milling. The resulting mixture was then heated at 500 °C for 120 min under a nitrogen atmosphere in a tube furnace. The N2 flow rate was 80 sccm min^−1^ at a 5 °C per min heating rate. The resulting product was denoted as SFC5.

#### 2.1.2. Preparation of the F-Doped Carbon Material (FC5)

The PVDF was subjected to a heat treatment at 500 °C for 120 min in a tube furnace filled with nitrogen. The obtained material was denoted as FC5.

#### 2.1.3. Preparation of the S-Doped Fluorine Carbon Material (FC5-S5)

The mixture of obtained FC5 and sublimed sulfur at a mass ratio of 1:2 was heated to 500 °C at a heating rate of 5 °C min^−1^ and maintained at 120 min in a tube furnace with a nitrogen flow rate of 80 sccm min^−1^. The obtained product was denoted as FC5-S5.

### 2.2. Materials Characterization

The morphological structure and texture of the prepared materials were observed by scanning electron microscopy (SEM; Hitachi S-4800, Hitachi High-Tech, Tokyo, Japan) with an energy dispersive X-ray spectroscopy (EDS; ULTIM MAX 65, Oxford Instruments, Oxford, UK) detector and high-resolution transmission electron microscopy (HRTEM; FEI Tecnai TF20, FEI Company, Hillsboro, OR, USA). X-ray photoelectron spectroscopy (XPS; ESCALAB250Xi, Thermo Fisher Scientific Inc, Waltham, MA, USA) was used to detect the surface chemical species and bonding states of the samples. X-ray powder diffraction (XRD; BRUKER D8 ADVANCE, Bruker Corporation, Billerica, MA, USA) was used to analyze the phase composition and crystal structures of the samples. Raman spectroscopy (LABRAM ARAMIS, excitation line of 514 nm, HORIBA Jobin Yvon S.A.S., Paris, France) was used to examine the structure of the samples.

### 2.3. Electrochemical Testing

The electrode slurry was prepared by mixing the active material, acetylene black, and sodium carboxymethyl cellulose at a mass ratio of 7:2:1 in deionized water. This slurry was uniformly blade-coated onto Al foil using a doctor blade with a gap setting of 150 μm, and subsequently vacuum-dried at 90 °C for 12 h to fabricate the working electrodes. The electrochemical performance of electrode materials was evaluated using the CR2025 coin cells, which were assembled in an argon-filled glove box with water and oxygen contents of <0.01 ppm. Metal sodium foil was used as the counter electrode, glass fibers were used as the separator, and 1.0 M NaPF_6_ dissolved in ethylene carbonate (EC) and diethyl carbonate (DEC) (1:1 *v*/*v*.) mixture solution as the electrolyte. The areal mass loading of the active materials for the working electrode was controlled to be 1.5–1.8 mg cm^−2^.

The galvanostatic discharge–charge tests and rate performance tests were performed in a Land CT3002A cell test system (Wuhan Land Electronics Co., Ltd., Wuhan, China) at the specified current density and voltage range of 0.01–2.50 V (vs. Na^+^/Na). Cyclic voltammetry (CV) and electrochemical impedance spectroscopy (EIS) measurements were performed on a Solartron 1470E Cell Test electrochemical workstation (Solartron Analytical AMETEK America, Berwyn, PA, USA). The CV curves were obtained at a sweep rate of 0.25 mV s^−1^ within the voltage range of 0–2.5 V (vs. Na^+^/Na), whereas EIS was measured in the frequency range of 10^−1^–10^5^ Hz with an amplitude of 5 mV.

The diffusion coefficient of Na^+^ (D_Na+_) in the electrode was determined using the galvanostatic intermittent titration technique (GITT). The pulse time and rest time were set to 30 min and 120 min, respectively, with a polarization current of 50 mA g^−1^. All batteries underwent a resting period of 48 h prior to testing.

## 3. Results and Discussion

### 3.1. Synthesis and Characterization

**Figure 1:**
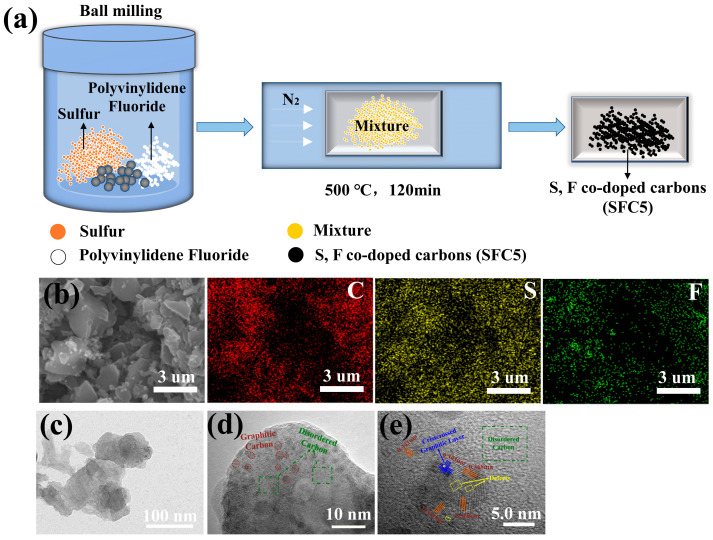
(**a**) Schematic of the synthesis of SFC5; (**b**) SEM image and EDS elemental mapping of SFC5; (**c**–**e**) TEM images of SFC5.

PVDF is an ideal precursor for preparing microporous carbon materials [24], and sublimed sulfur is generally used as a sulfur source to dope and/or infuse sulfur into matrices [28]. Therefore, the S-infused/S, F-codoped carbon material SFC5 was prepared by annealing a mixture of PVDF and sublimed sulfur under an N_2_ atmosphere at 500 °C, and the synthesis route of SFC5 is shown in Figure 1a. PVDF underwent pyrolysis during annealing, which was accompanied by the release of HF gas, and the carbonization product of PVDF was fluorine-doped microporous carbon [29]. Moreover, the sublimed sulfur reacted with the carbonization product of PVDF and infused into its microporous structure, forming the S-infused/S, F-codoped carbon SFC5.

The morphological features of the PVDF and heat-treated products were observed by SEM, and the images are shown in Figure S1. PVDF is a sphere with an average diameter of ~150 nm (Figure S1a), but it changes into irregular bulk carbon after carbonation (Figure S1b–d). Figure 1b presents the EDS element mappings of SFC5, confirming its composition of carbon (C), sulfur (S), and fluorine (F). The spatial overlap between S/F and C distributions further verifies that PVDF has undergone carbonation and that S and F were successfully codoped into the carbon framework. The TEM images are shown in Figure 1c–e to verify the texture of SFC5. A low-magnification TEM image of SFC5 is shown in Figure 1c, which reveals that SFC5 has a sheet structure. Its magnified local image is shown in Figure 1d, where obvious dark areas marked by red circles can be found. To further ascertain the structure of these dark areas, high-resolution TEM was applied, and the image is shown in Figure 1d. Lattice fringes representing different lattice spacings are found in the dark area, indicating that the dark areas are crystalline carbon and that the surrounding material is amorphous carbon. The dark areas contain discontinuous microcrystalline graphite, crisscrossing graphitic layers, and defects. These results demonstrate that the prepared SFC5 is a short-range ordered amorphous carbon material. Notably, the maximum interlayer spacing of SFC5 (0.368 nm, Figure 1e) is larger than that of graphite (0.34 nm), implying that the interlayer spacing was expanded by introducing heteroatoms.

**Figure 2:**
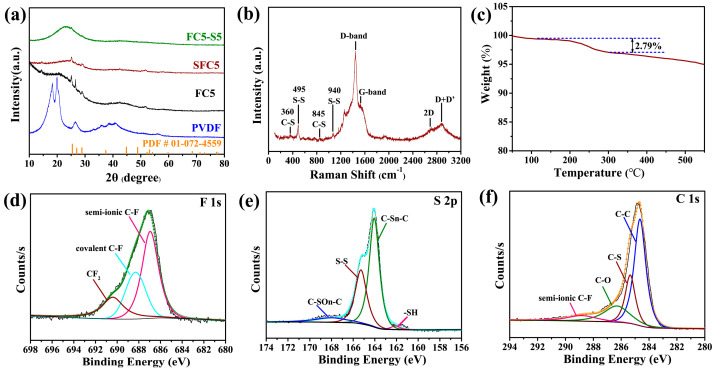
(**a**) XRD patterns of SFC5, FC5-S5, FC5, and PVDF; (**b**) Raman spectrum of SFC5; (**c**) TGA curve of SFC5; (**d**–**f**) high-resolution XPS spectra of SFC5.

The nitrogen adsorption–desorption isotherms of the samples are shown in Figure S2. All three samples (SFC5, FC5, and FC5-S5) display characteristic Type I isotherms, confirming their microporous nature [24,29]. However, the micropore features of SFC5 (Figure S2a) and FC5-S5 (Figure S2c) are slightly different from those of FC5 (Figure S2b). SFC5 and FC5-S5 have smaller volumes. The corresponding micropore size distributions are shown in the inset. The micropore sizes of SFC5, FC5, and FC5-S5 are distributed mainly at ~0.5 nm, ~0.8 nm, and ~1.1/1.3 nm, respectively. According to the BET method, the specific surface areas of SFC5 and FC5-S5 are 391.45 m^2^ g^−1^ and 245.38 m^2^ g^−1^, whereas the FC5 sample had a relatively large specific surface area of 774.15 m^2^ g^−1^. These differences among SFC5, FC5-S5, and FC5 can be attributed to the incorporation of sulfur into the micropores of the PVDF-derived carbons. The X-ray diffraction patterns of the samples are shown in Figure 2a. The XRD pattern of FC5 displays two broad diffraction peaks located at approximately 2θ = 23° and 43°, corresponding to the (002) and (100) crystal planes of graphite [29], respectively. Furthermore, three sharp diffraction peaks are observed within the 2θ range of 25°–30°, corresponding to silicon impurities as they align well with the standard silicon reference pattern (PDF # 01-072-4559). These silicon impurities originate from quartz tube corrosion caused by HF gas generated during PVDF pyrolysis [30]. Although residual traces of these three peaks can still be detected in SFC5 and FC5-S5, their intensities are substantially diminished. This observation indicates that the sulfur doping process and incorporated sulfur may obscure the silicon impurity signals in XRD analysis. Despite the presence of sulfur in SFC5, some sulfur forms covalent bonds with carbon, resulting in the formation of sulfur-doped carbon. The remaining amount of elemental sulfur was confirmed through XPS and TGA (Figure 2c) characterization. However, the content of elemental sulfur (2.79 wt%) in SFC5 is insufficient to be detected by X-ray diffraction (XRD); therefore, no characteristic peaks were observed.

XPS analysis was conducted to investigate the surface chemical species of FC5, SFC5, and FC5-S5. As expected, FC5, SFC5, and FC5-S5 contain C, F, and O, and SFC5 and FC5-S5 also contain S (Figure S3). The detailed atom ratios of the elements on the surfaces of FC5, FC5-S5, and SFC5 are shown in Table S1. The concentration of F in SFC5 (measured by XPS) is 9.54 at.% because the optimal pyrolysis temperature preserves a considerable number of C–F bonds in PVDF, whereas FC5 has a lower fluorine content of 8.66 at.%, suggesting that the sulfur in SFC5 may contribute to stabilizing the C–F bond during pyrolysis. However, the fluorine content sharply decreased to 2.34 at.%, and the sulfur content was 8.01 at.% in FC5-S5 prepared by a two-step method because of the poor thermal stability of the C–F bond. The high-resolution XPS deconvolutions of F 1s, S 2p, and C 1s peaks for SFC5 are shown in Figure 2d–f. The F 1s XPS spectrum of SFC5 (Figure 2d) was deconvoluted into three distinct peaks at 686.8, 688.3, and 690.4 eV, corresponding to semi-ionic C–F bonds, covalent C–F bonds, and CF_2_ functional groups, respectively [31,32]. The S 2p spectrum (Figure 2e) can be deconvoluted into four distinct peaks at 162.1, 164.1, 165.3, and 168 eV, corresponding to reduced sulfur (-SH), C–S_n_–C (n = 1 or 2) bonds (from thiophene-type sulfur), S-S bonds, and oxidized sulfur (C-SO_n_-C) [33,34], respectively. This observation confirms the successful incorporation of sulfur into SFC5. The C 1s spectrum (Figure 2f) can be divided into four peaks at 284.6, 285.3, 286.3, and 288.8 eV which were assigned to C–C, C–S, C–O/C=O, and semi-ionic C–F bonds, respectively, demonstrating the successful introduction of S and F atoms into the carbon framework of SFC5. The high-resolution XPS deconvolutions of the S 2p, F 1s, and C 1s peaks for FC5-S5 (Figure S3d–f), as well as the F 1s and C 1s peaks for FC5 (Figure S3b,c), are provided as controls for SFC5. In contrast to SFC5, which exhibited three peaks, the F 1s spectra of FC5 (Figure S3b) and FC5-S5 (Figure S3e) were deconvoluted into only two peaks, corresponding to semi-ionic C-F bonds and covalent C-F bonds, respectively. Notably, the peak associated with CF_2_ functional groups is absent in their F 1s spectra. Moreover, the area of each individual peak in these samples is significantly smaller than that in SFC5. In the high-resolution C 1s XPS spectra of FC5 (Figure S3c) and FC5-S5 (Figure S3f), peaks attributed to C–C, C–O/C=O, and semi-ionic C–F bonds are also observable. For the semi-ionic C–F bonds, a decrease in intensity and an upshift in binding energy are evident. These results are consistent with the fluorine concentrations in SFC5, FC5-S5, and FC5, further verifying that the sulfur in SFC5 contributes to stabilizing the C–F bond during pyrolysis. The high-resolution S 2p XPS spectrum of FC5-S5 (Figure S3d) is similar to that of SFC5 (Figure 2e), displaying four distinct peaks corresponding to reduced sulfur (-SH), C–Sₙ–C (n = 1 or 2) bonds, conjugated −C=S– bonds (from thiophene-type sulfur), and oxidized sulfur –SOₙ^−^), respectively. This confirms the successful incorporation of sulfur into FC5-S5.

The thermogravimetric analysis (TGA) of SFC5 was conducted under an N_2_ flow, as shown in Figure 2c. There are three temperature ranges, 0–100 °C, 200–300 °C, and above 300 °C, where mass loss can be observed, which correspond to the processes of water evaporation, elemental sulfur evaporation, and decomposition of residual volatile impurities in SFC5, respectively. Notably, the sulfur content in SFC5 was 2.79 wt%.

The Raman spectrum of SFC5 is shown in Figure 2b. Distinct peaks at 360 and 820 cm^−1^ are attributed to the stretching vibration and deformation of the C–S bonds, and those at 494 and 938 cm^−1^ are assigned to the stretching vibration of the S–S bonds [19,35], indicating that the sulfur atoms are bonded to the carbon atoms. As anticipated, Raman spectra of FC5-S5 retain the characteristic C-S and S-S vibrational modes observed in SFC5, albeit with markedly reduced intensity. In contrast, these sulfur-related peaks are completely absent in FC5, consistent with its non-sulfurized nature. In addition, four prominent characteristic peaks related to the structure of carbon are observed at 1420, 1530, 2700, and 2870 cm^−1^, corresponding to the D, G, 2D, and D + D′ bands, respectively [31]. Both FC5-S5 and FC5 exhibit the typical D-band (1350 cm^−1^) and G-band (1580 cm^−1^) characteristic peaks of carbon materials. Notably, the D-band in SFC5 shows a significant upshift to 1420 cm^−1^ from the standard 1350 cm^−1^ position, demonstrating substantially higher defect density compared with FC5-S5 and FC5. Among these peaks, the D-band and D + D′ were used to reflect the density of defects and edges for carbon materials (the degree of disorder), whereas the G-band and 2D peak are indicators of the degree of graphitization. Moreover, the peak intensity ratio of the D-band and G-band (I_D_/I_G_) also reflects the degree of disorder of the carbon materials [36]. The I_D_/I_G_ values for SFC5, FC5-S5, and FC5 are 1.775, 0.975, and 0.969, respectively. These findings further verify that SFC5 is a carbon material with abundant defects. In contrast to SFC5, the peak shape and intensity of 2D and D + D′ bands for FC5 and FC5-S5 display obvious variations, coupled with lower I_D_/I_G_ ratios. These results indicate that FC5 and FC5-S5 contain significantly fewer defects and greater stacking thicknesses compared with SCF5. The abundant defects and thin stacking thickness of SFC5 together contributed to its outstanding sodium storage performance.

### 3.2. Electrochemical Performance Evaluation

**Figure 3:**
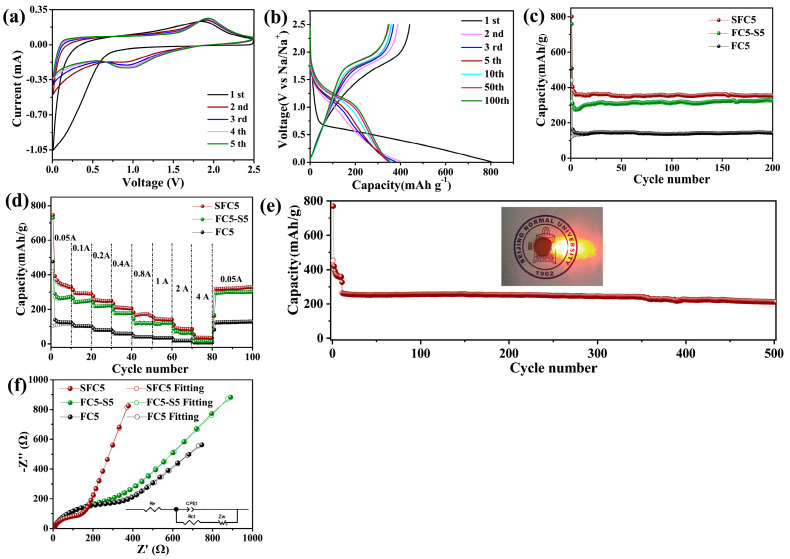
(**a**) CV curves of the SFC5 composite electrode; (**b**) Galvanostatic discharge–charge curves of SFC5; (**c**) cycling performance of the SFC5, FC5, and FC5-S5 composite electrodes at 0.05 A g^−1^; (**d**) rate capability of the SFC5, FC5, and FC5-S5 composite electrodes; (**e**) long-term cycling stability over 500 cycles with the SFC5 composite at 0.4 A g^−1^ (the inset shows the LED lights illuminated by the coin cell); (**f**) Nyquist plots of the SFC5, FC5, and FC5-S5 composite electrodes (the inset shows the equivalent circuit model used for fitting the experimental impedance).

The electrochemical sodium storage mechanisms of the prepared samples were investigated by cyclic voltammetry (CV) and galvanostatic discharge–charge (GDC) tests. Figure 3a shows the initial five-cycle CV curves of SFC5, where the voltage range is 0.01–2.5 V (vs. Na^+^/Na) and the scan rate is 0.25 mV s^−1^. For the cathode scan, an obvious increase in the cathode current occurs in the voltage range of 0.75–0 V (vs. Na^+^/Na), representing the formation process of the solid electrolyte interface (SEI) and the sodium storage reaction of hard carbon and S during the initial cathode scan, and two prominent cathode peaks appear at 1.0 V (vs. Na^+^/Na) and approach 0 V (vs. Na^+^/Na), which correspond to the sodium storage reactions of S and hard carbon, respectively. The CV curve corresponds to the charge–discharge curves shown in Figure S6. For SFC5, the initial discharge curve shows a sloping plateau from 0.75–0 V (vs. Na^+^/Na). For the anode scan, an apparent anodic peak appeared at ~1.8 V (vs. Na^+^/Na) in the initial CV curve, and it persisted during the subsequent scan. This anode peak represents the desodiation reaction of sulfide, and its repeatability implies high reversibility of the electrode reaction. The corresponding charge curves are shown in Figure S6a. A sloping plateau and a voltage plateau are observed at ~1.8 V (vs. Na^+^/Na). The sloping plateau represents the desodiation process of hard carbon, and the voltage plateau is consistent with the anode peak in the CV curves. As shown in Figures S5b and S6b, the CV curves and charge–discharge curves of FC5-S5 are analogous to those of SFC5 in shape, but a considerable difference in intensity is observed because SFC5 contains more S than FC5-S5. FC5 has not been sulfurized, so its CV and charge–discharge curves are considerably different from those of SFC5 and FC5-S5. The cathode/anode current can be observed in the CV curves only in a voltage range, and the corresponding sloping plateau can be observed in the charge–discharge curves, as shown in Figures S5a and S6b, which represent the sodiation/desodiation of hard carbon.

The GDC profiles of the samples produced at a current density of 50 mAh g^−1^ are shown in Figure S6. The initial discharge curve of SFC5 shows a relatively gentle sloping plateau and a discharge capacity of 800.7 mAh g^−1^ with a coulombic efficiency of 55.05%, and the corresponding charge curve comprises a sloping plateau and a voltage plateau. The discharge curves exhibit a sloping plateau and a voltage plateau in the subsequent cycles, the coulombic efficiency increases with increasing cycle number, and an impressive value of 99.6% is achieved in the fifth cycle. The irreversible capacity loss during the initial cycle is attributed to SEI formation, whereas the increase in coulombic efficiency indicates that the SEI is passivating and that its structure gradually tends to be stable. As shown in Figure S6b, FC5-S5 has an initial discharge capacity of 758.9 mAh g^−1^ and an ICE of 42.2%, which are lower than those of SFC5 because of the lower degree of sulfuration of FC5-S5 by the two-step method. FC5, as shown in Figure S6b, delivers the lowest initial discharge capacity of 507.7 mAh g^−1^ and ICE of 24.1% because it has not been sulfurized. These results demonstrate that the sodium storage performance of PVDF-derived carbon can be dramatically improved by synchronously implementing a sulfuration process during the pyrolysis of PVDF.

The electrodes comprising different samples are subjected to 200 cycles in a half-cell architecture at a current density of 50 mAh g^−1^ to evaluate the cycling stability of the prepared materials. As shown in Figure 3b, the SFC5, FC5-S5, and FC5 electrodes display obvious capacity fading during the initial several cycles, with coulombic efficiencies lower than 99%, but their reversible capacities tend to be stable, and their coulombic efficiencies reach over 99.0% after the initial five cycles of activation. The reversible capacities of SFC5, FC5-S5, and FC5 are 358.8, 324.2, and 267.4 mAh g^−1^ after the initial five cycles of electrode activation, respectively, and their capacity retention rates are 93.3%, 91.7%, and 86.2%, respectively, after 200 cycles. The sodium storage performance of PVDF-derived carbon undergoing sulfurization is considerably superior to that of PVDF-derived carbon without sulfurization in terms of capacity or cycling stability, and the SFC5 prepared by one-step pyrolysis and synchronous sulfurization is slightly better than the FC5-S5 prepared by stepwise pyrolysis followed by sulfurization. The substantial difference in capacity between PVDF-derived carbon with and without sulfurization originates from the plateau capacity observed in the discharge–charge curves of SFC5 and FC5-S5, which is associated with the sodium storage reaction of S. The difference in capacity between SFC5 and FC5-S5 is attributed to the S content of SFC5 being higher than that of FC5-S5.

The outstanding rate performances of the SFC5, FC5-S5, and FC5 electrodes are shown in Figure 3c. SFC5 delivers reversible capacities of 326, 295, 250, 210, 170, 140, 90, and 40 mAh g^−1^ at current densities of 0.05, 0.1, 0.2, 0.4, 0.8, 1.0, 2.0, and 4.0 A g^−1^, respectively, and the capacity recovers to 325 mAh g^−1^ when the current density returns to 0.05 A g^−1^. The outstanding rate performance of SFC5 originates from the high concentration of S, F codoping, and the intrinsically high electron/ion conductivity of the carbon matrix. These electrode properties are beneficial for improving the electrode reaction kinetics. To further evaluate its superior sodium storage performance at high current density, SCF was subjected to a long-term cycling test at 0.4 A g^−1^ (as shown in Figure 3d). After 500 cycles, the reversible capacity was 212 mAh g^−1^, with a capacity decay rate of 0.00165% per cycle. As illustrated in the insert in Figure 3d, the cell can easily illuminate a red LED, demonstrating the practicability of the SFC5 applied in SIBs.

The electrochemical impedance spectroscopy (EIS) spectra of the FC5, FC5-S5, and SFC5 electrodes after three cycles at 50 mA g^−1^ are displayed in Figure 3e. In the high/middle-frequency region, all three electrodes exhibit a single semicircle in their impedance spectrum, and a sloping straight line is observed in the low-frequency range. To analyze these impedance spectra, we propose a straightforward equivalent circuit model, as illustrated in the insert, where Re represents the resistance of the electrode and electrolyte, and Rct, which is defined as the charge transfer impedance, is related to the electrode reaction kinetics. The SFC5 electrode has a lower Rct (182.4 Ω) than FC5 (344.7 Ω) and FC5-S5 (216.8 Ω), which is in accordance with the results of the rate performance tests of these three electrodes. The root reason for this phenomenon is that high concentrations of S, F codoping bring abundant heteroatom active sites to pyrolytic carbon and improve the electron/ion conductivity of the electrode.

### 3.3. Quantitative Analysis of the Electrode Reaction Kinetics of SFC5

**Figure 4:**
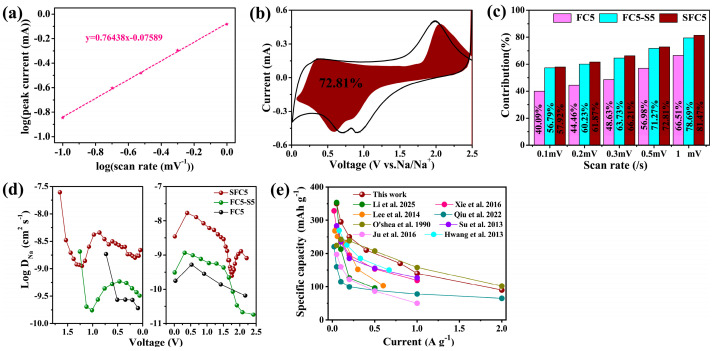
(**a**) Plots of log (i) versus log (ν) corresponding to the oxidation peak of the SFC5 composite electrode. (**b**) Pseudocapacitance contribution (red region) of the SFC5 composite electrode at a sweep rate of 0.5 mV s^−1^. (**c**) Pseudocapacitance contribution of FC5, FC5-S5, and SFC5 at various sweep rates. (**d**) D_Na+_ versus the potential of SFC5, FC5-S5, and FC5 composite electrodes during discharge and charge. (**e**) The rate capability of the SFC5 composite electrode compared with that of references involving various doped carbon hybrid electrodes for SIBs [13,29,30,31,32,33,34,35].

There are three typical types of charge storage mechanisms for electrode materials: diffusion-controlled faradaic battery behavior, surface-controlled faradaic pseudocapacitive behavior, and surface-controlled nonfaradaic capacitive behavior. Among them, surface-controlled pseudocapacitive and capacitive behaviors feature a high rate of charge storage and are closely related to the rate performance of electrodes. The peak current has a weak relationship with the sweep rate ν in the CV tests, as shown in Formula (1). Both a and b are adjustable parameters, and the b value is an indicator used to distinguish the type of charge storage. The b value is determined by the slope of the plot of $\log{(i}_{p})$ versus log(ν), as shown in Formula (2). There are two well-defined conditions, b = 0.5 and b = 1.0, which represent diffusion-controlled and surface-controlled charge storage processes, respectively. However, the b value generally lies in the range of 0.5–1.0 for battery materials, indicating that these two types of charge storage mechanisms coexist. The CV of the SFC5 electrode was conducted at different sweep rates ranging from 0.1 to 1 mV s^−1^, and the results are shown in Figure S7a. The corresponding plots of $\log{(i}_{p})$ versus log(ν) are shown in Figure 4a, and the correlation function is y = 0.76438x − 0.07589, so the b value is 0.76438, implying that the sodium storage capacity of SFC5 is composed of the diffusion-controlled bulk redox reaction capacity contribution and surface-controlled pseudocapacitance contribution.

(1) $$I_{p}=a\nu^{b},$$

(2) $$\log{(i}_{p})=\log(a)+b\log\left(\nu \right),$$

Because the electrode reaction of SFC5 combines a diffusion-controlled bulk redox reaction and surface-controlled pseudocapacitance, Formula (3) can be employed to assign the current response to the corresponding charge storage process, where $i\left(V\right)$ is the current at the selected voltage, ν is the voltage sweep rate of the CV, and $k_{1}$ and $k_{2}$ are correlation coefficients. $k_{1}\nu$ and $k_{2}\nu^{1/2}$ are associated with the current response related to the diffusion-controlled reaction and surface-controlled pseudocapacitance, respectively. The k_1_ and k_2_ values are determined by the slope and y-axis intercept point of Formula (4), respectively. The k_1_ value in the voltage range of the CV scan can be acquired by the abovementioned method, and the corresponding $k_{1}\nu$, which represents the current response related to the diffusion-controlled pseudocapacitance process, can be obtained and plotted versus V. The ratio of the area enclosed by the $k_{1}\nu$–V curve to the area enclosed by the CV curves represents the surface-controlled pseudocapacitance contribution. Figure 4b shows the pseudocapacitance contribution of SFC5 at a sweep rate of 0.5 mV s^−1^, which is 72.81%, which is consistent with a high rate performance. Figure 4c depicts the ratio of the surface-controlled pseudocapacitance contributions of the SFC5, FC5-S5, and CF5 electrodes at various sweep rates. The SFC5 electrode clearly has the highest pseudocapacitance contribution, followed by FC5-S5 and then FC5. The reason is that a high concentration of S, F codoping introduces more heteroatomic sites and defects into SFC5 and FC5-S5, which favor the surface-controlled pseudocapacitance process.

(3) $$i\left(V\right)=k_{1}\nu +k_{2}\nu^{1/2},$$

(4) $$i\left(V\right)/\nu^{1/2}=k_{1}\nu^{1/2}+k_{2}$$

To elucidate the outstanding sodium storage performance of SFC5, the galvanostatic intermittent titration technique (GITT) was employed to quantitatively analyze the Na^+^ diffusion coefficient (D_Na+_) inside the electrodes. The cell for GITT tests was discharged/charged at a current density of 50 mA g^−1^ for 0.5 h and then relaxed for 2.0 h, and this process was repeated until the cutoff voltage was reached. The results of the GITT measurements for SFC5 are shown in Figure S8. The plots of D_Na+_ versus time during discharge–charge for the three electrodes are shown in Figure 4d. SFC5 exhibits the highest D_Na+_ because it has higher S and F codoping concentrations than FC5-S5 and FC5. The high D_Na+_ contributes to the outstanding sodium storage performance of SFC5, including its high rate performance and long cycle life.

Compared with other reported heteroatom-doped carbon materials, as shown in Figure 4e [15,37,38,39,40,41,42,43], SFC5 has better electrochemical sodium storage performance, which is attributed to the following two aspects: (1) PVDF-derived carbon is an ideal carbon matrix because the carbonization product of PVDF is a fluorine-doped carbon material with a microporous structure. (2) The proper material preparation strategy for SFC5 involves one-step pyrolysis and synchronous sulfurization to achieve a high concentration of in situ doping of F and S synchronously, and the infusion of sulfur into the micropores of the carbon matrix. The high concentration of S and F codoping introduced many heteroatom sites and defects into the carbon matrix and expanded the interlayer space of the carbon, and the incorporation of sulfur into the micropores of the carbon matrix synergistically endowed SFC5 with outstanding sodium storage performance.

**Figure 5:**
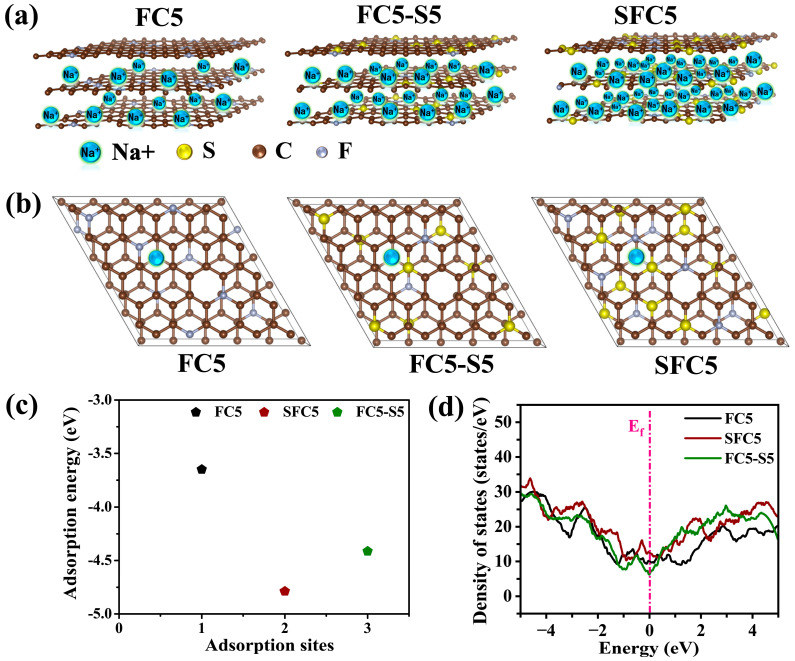
(**a**) Graphical illustration of the sodium storage capacity of FC5, FC5-S5, and SFC5; (**b**) structural model used to calculate the Na^+^ adsorption energy of FC5, FC5-S5, and SFC5; (**c**) Na^+^ adsorption energy of FC5, FC5-S5, and SFC5; (**d**) density of states of FC5, FC5-S5, and SFC5.

Furthermore, the Na^+^ adsorption energy and density of states (DOS) for SFC5, FC5-S5, and FC5 were obtained via density functional theory (DFT)-based first principles. Figure 5a shows the Na storage schematics of FC5, FC5-S5, and SFC5, and the models used in the theoretical simulation were constructed in accordance with the element ratios obtained from the XPS results, as shown in Figure 5b. Figure 5c shows that the Na^+^ adsorption energies (ΔE_a_) for SFC5, FC5-S5, and FC5 are −4.78 eV, −4.61 eV, and −2.91 eV, respectively. Notably, the Na^+^ adsorption energy of S, F codoping of SFC5 and FC5-S5 is much lower than that of FC5, and that of SFC5 is the lowest, which signifies that a high concentration of S, F codoping is beneficial for Na^+^ adsorption by carbon materials. Moreover, the DOS used to reflect the electrical conductivity of the materials was calculated, as shown in Figure 5d. SFC5 and FC5-S5 display a higher DOS near the Fermi level than does FC5, implying that the electron conductivity is enhanced with increasing concentrations of S, F codoping. The results of theoretical computations indicate that a high concentration of S, F codoping can dramatically reduce the Na^+^ adsorption energy and increase the electronic conductivity of PVDF-derived carbon.

## 4. Conclusions

In summary, high-concentration S-infused/S, F-codoped PVDF-derived carbon was prepared by one-step carbonization of PVDF and a synchronous sulfurization process as a high-performance anode for sodium-ion batteries. The high concentration of S, F codoping introduces abundant defects and heteroatom sites into the PVDF-derived carbon and expands the interlayer spacing of the carbon, and the infused S provides additional sodium storage capacity. In addition, electrode kinetic analysis and theoretical computation revealed that high-concentration S-infused/S, F-codoped PVDF-derived carbon has increased ionic and electronic conductivity as well as decreased Na^+^ adsorption energy, resulting in its outstanding sodium storage performance. The results of this work highlight that sulfur- and fluorine-codoped carbon materials hold great promise as anode materials for sodium-ion batteries.

## Data Availability

The original contributions presented in this study are included in the article/Supplementary Materials. Further inquiries can be directed to the corresponding author.

## Supplementary Materials

The following supporting information can be downloaded at: https://www.mdpi.com/article/10.3390/ma18174018/s1, Figure S1: SEM images of (a) PVDF, (b) SFC5, (c) FC5, (d) FC5-S5; Figure S2: Nitrogen adsorption–desorption isotherms (a) SFC5, (b) FC5, and (c) FC5-S5. Insets show the corresponding pore size distribution curves; Figure S3: (a) XPS survey spectra of SFC5, FC5-S5, and FC5; (b,c) High-resolution XPS spectra of FC5; (d–f) High-resolution XPS spectra of FC5-S5; Figure S4: Raman spectra of (a) FC5; (b) FC5-S5; Figure S5: CV curves of (a) FC5; (b) FC5-S5 composite electrodes; Figure S6: Galvanostatic discharge–charge curves of (a) FC5 and (b) FC5-S5 composite electrodes; Figure S7: CV curves at different sweep rates (a) SFC5, (b) FC5 and (c) FC5-S5 composite electrodes; Figure S8: Galvanostatic intermittent titration (GITT) curve for SFC5 composite electrode; Table S1: The atom ratio of elements on the surface of FC5, FC5-S5, and SFC5; Table S2: Electrochemical impedance parameters of FC5, FC5-S5, and SFC5. The theoretical calculation parameters and their references [44,45,46,47] are cited in the Supplementary Materials.
