# Supplementary material for: An S-Infused/S, F-Codoped PVDF-Derived Carbon as a High-Performance Anode for Sodium-Ion Batteries
Source: Materials (Basel). 2025 Aug 27;18(17):4018. doi: 10.3390/ma18174018 (PMC12429498; doi:10.3390/ma18174018)
Supplement: Supplementary file 1 [file materials-18-04018-s001.zip › materials-3772645-supplementary.pdf]

Supporting information

An S-infused/S, F-Codoped PVDF-Derived Carbon as a High-Performance Anode for Sodium-Ion Batteries

Jianjiao Wang, Qian Zhang, Pengyu Han, Jiakun Luo, Kui-Qing Peng*.

Key Laboratory of Multiscale Spin Physics, Ministry of Education, Beijing Key Laboratory of Energy Conversion and Storage Materials, School of Physics and Astronomy, Beijing Normal University, Beijing, 100875, PR China

* Correspondence: kq_peng@bnu.edu.cn

Figures:

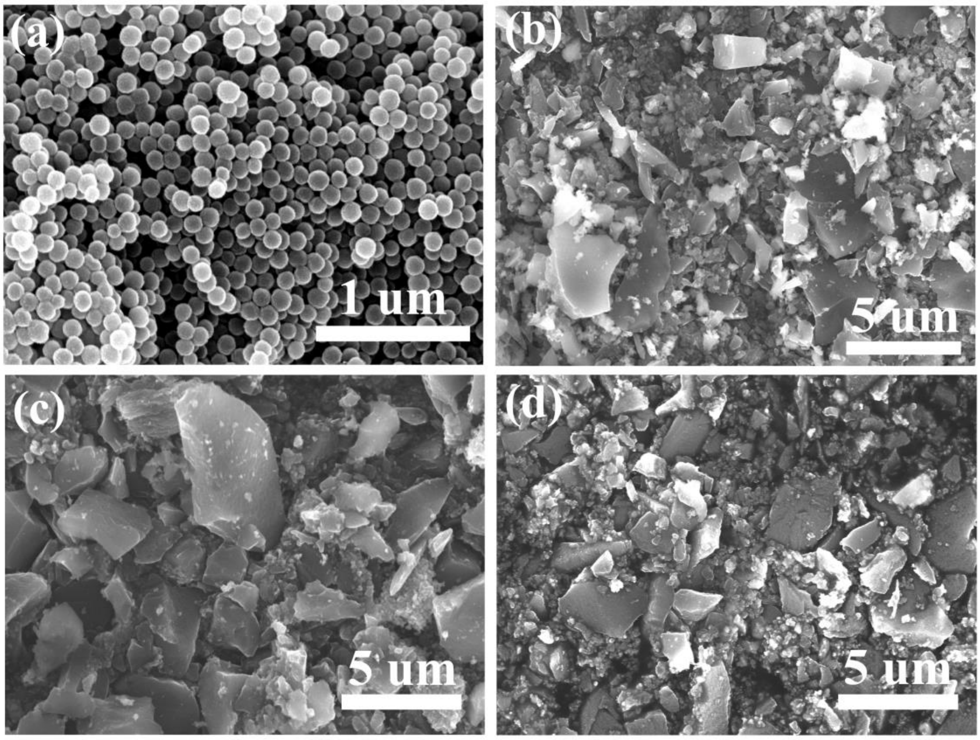

Figure S1. SEM images of a) PVDF, b) SFC5, c) FC5, d) FC5-S5.

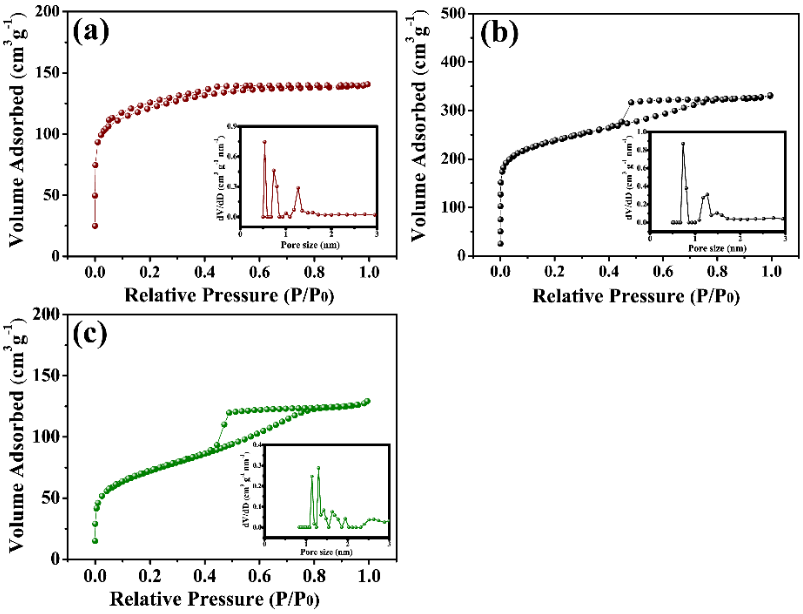

Figure S2. Nitrogen adsorption-desorption isotherms (a) SFC5, (b) FC5, and (c) FC5-S5. Insets show the corresponding pore size distribution curves.

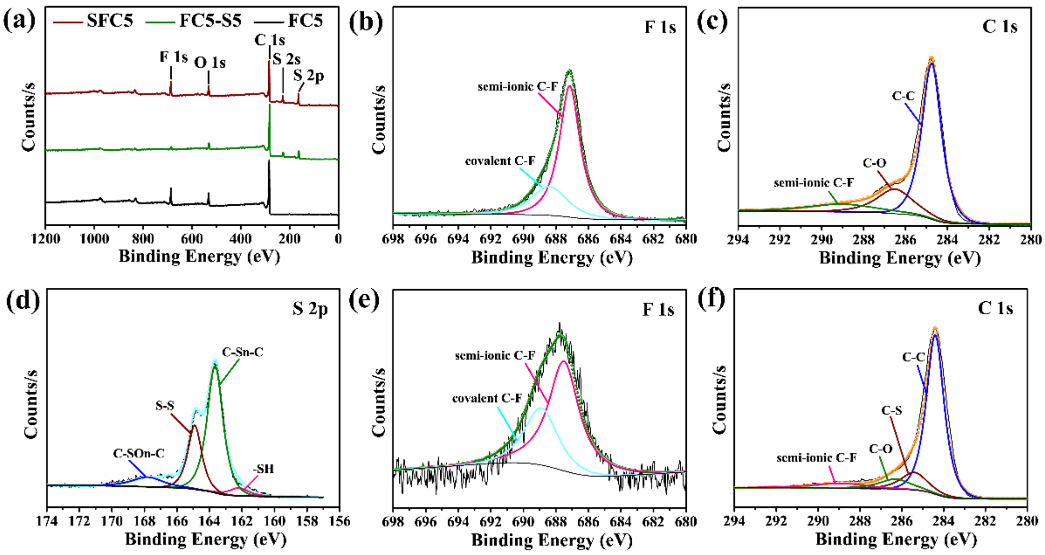

Figure S3. (a) XPS survey spectra of SFC5, FC5-S5 and FC5; (b-c) High-resolution XPS spectra of FC5; (d-f) High-resolution XPS spectra of FC5-S5.

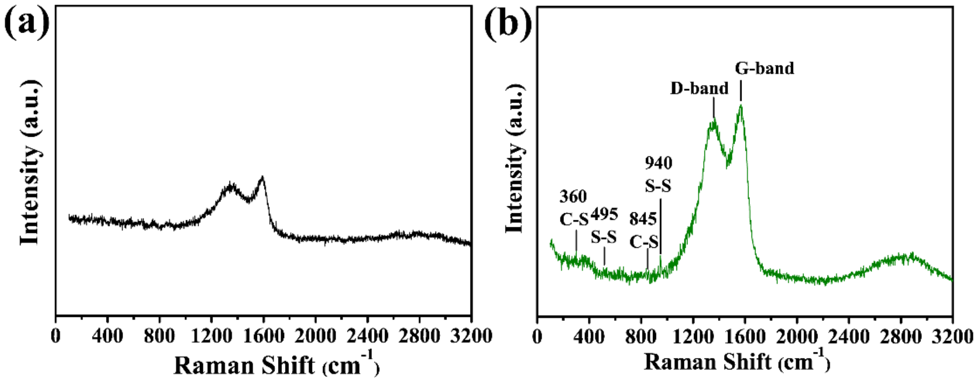

Figure S4. Raman spectra of a) FC5; b) FC5-S5.

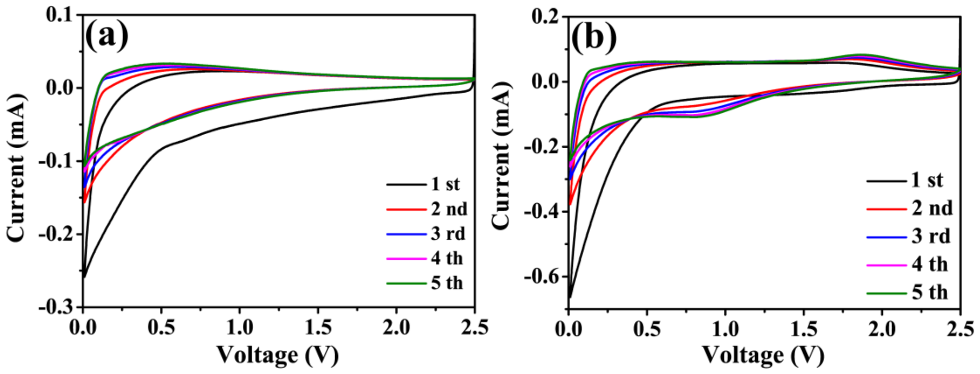

Figure S5. CV curves of a) FC5; b) FC5-S5 composite electrode.

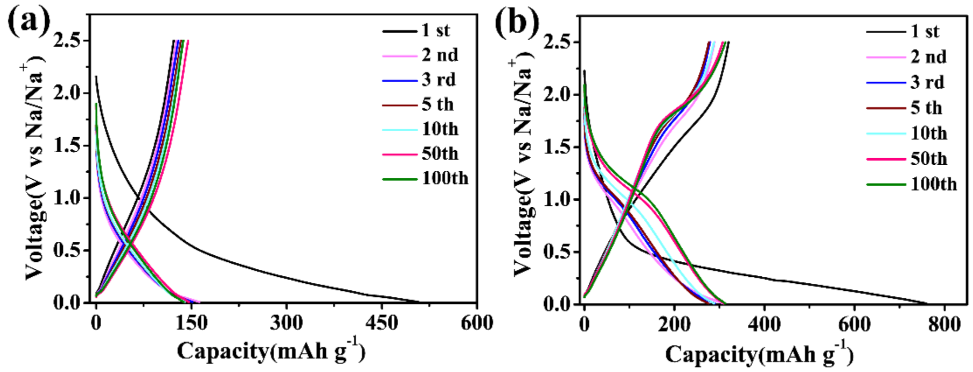

Figure S6. Galvanostatic discharge-charge curves of a) FC5 and b) FC5-S5 composite electrode.

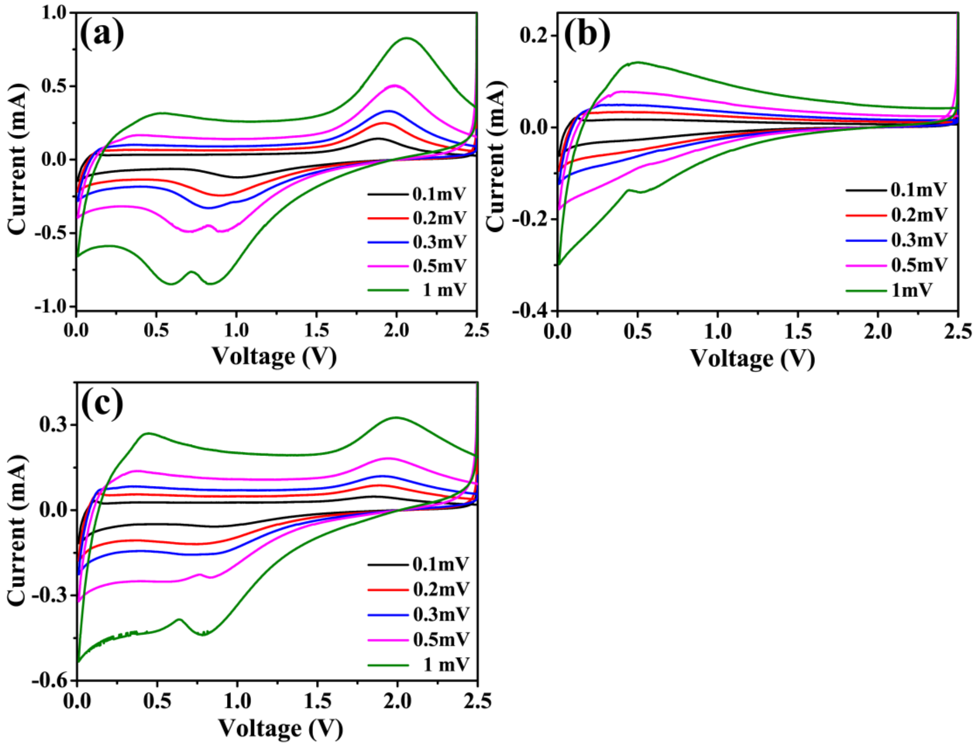

Figure S7. CV curves at different sweep rates a) SFC5, b) FC5 and c) FC5-S5 composite electrode.

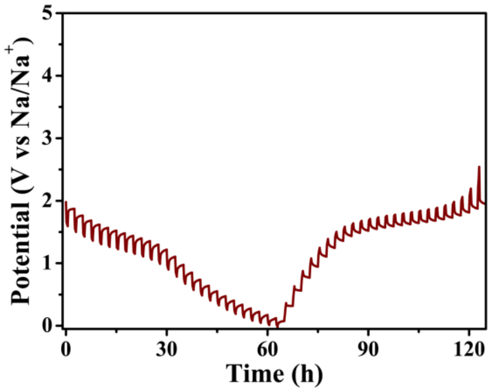

Figure S8. Galvanostatic intermittent titration (GITT) curve for SFC5 composite electrode.

Table:

Table S1. The atom ratio of elements on the surface of FC5, FC5-S5 and SFC5

Samples	Element content (at. %)			
	C	S	F	O
**FC5**	82.45	0	8.66	8.9
**FC5-S5**	83.91	8.01	2.34	5.74
**SFC5**	71.56	10.11	9.54	8.8

Table S2. Electrochemical impedance parameters of FC5, FC5-S5 and SFC5.

	FC5	FC5-S5	SFC5
**Rct (Ω)**	344.7	216.8	182.4

Theoretical calculations:

The first-principles calculations are performed on the basis of density functional theory (DFT) implemented in the VASP[44] package, and the electronic states are expanded using the projector augmented wave (PAW)[45] approach with a cutoff of 500 eV. The energy convergence criterion is 10^{-5} eV, and the maximal residual force is smaller than 10^{-2} eV $Å^{-1}$. The exchange–correlation energy is described by the modified Perdew–Burke–Ernzerhof (PBE) functional[46] with semiempirical corrections of the DFT-D3 method based on the general gradient approximation (GGA). A 5 × 5 × 2 graphite-like carbon supercell with defects is calculated. The Brillouin zone is sampled with a 7 × 7 × 9 Monkhorst mesh, and the force convergence threshold is 0.05 eV/Å. The density of states calculations were performed with density functional theory (DFT) by combining the Vienna ab initio simulation package (VASP) with the postprocessing VASPKIT[47] package.
